# Three-Dimensional Bioprinting of Regenerative Cartilage Constructs with Directional Ionically Derived Stiffness Gradients

**DOI:** 10.3390/jfb16120451

**Published:** 2025-12-03

**Authors:** Maryam Hosseini, Angeliki Dimaraki, Gerjo. J. V. M. van Osch, Lidy E. Fratila-Apachitei, Pedro J. Díaz-Payno, Amir A. Zadpoor

**Affiliations:** 1 Department of Biomechanical Engineering, Faculty of Mechanical Engineering, Delft University of Technology, 2628 CD Delft, The Netherlandsg.vanosch@erasmusmc.nl (G.J.V.M.v.O.); e.l.fratila-apachitei@tudelft.nl (L.E.F.-A.); 2 Department of Orthopedics and Sports Medicine, Erasmus MC, University Medical Center, 3015 CN Rotterdam, The Netherlands; 3 Department of Otorhinolaryngology, Head and Neck Surgery, Erasmus MC, University Medical Center, 3015 CN Rotterdam, The Netherlands; 4 IMDEA Materials Institute, 28906 Getafe, Madrid, Spain; 5 Department of Orthopedics, Leiden University Medical Center, 2333 ZG Leiden, The Netherlands

**Keywords:** 3D bioprinting, stiffness gradient, tissue engineering, articular cartilage, human chondrocytes

## Abstract

Tissue engineering approaches for cartilage tissue regeneration are expanding to include the complex features of the tissue, such as the biological and mechanical gradients. Many of these approaches are, however, based on the use of multiple biomaterials or concentrations, and crosslinking methods that make it difficult to integrate and control the properties of the resulting scaffolds. In this study, a 3D bioprinted scaffold with a stiffness gradient was fabricated by using a single biomaterial type and concentration combined with a directional ionic crosslinking method. The scaffolds revealed a gradient in stiffness from 39.8 ± 6.6 kPa at the top to 60.6 ± 10.9 kPa at the bottom of the scaffolds. Live/dead analysis of human chondrocytes embedded in the scaffolds showed no negative effects of the stiffness gradient on cell viability over 28 days. The induced stiffness gradient led to a gradient in cell density and sulfated glycosaminoglycan deposition in the bioprinted tissue constructs with enhanced values in the softer top region of the scaffolds as compared to the stiffer bottom part. This study showed a novel method to generate scaffolds with stiffness gradients from a single biomaterial and indicates that such scaffolds could be used to spatially regulate the behavior of chondrocytes and the associated deposition of the cartilage matrix.

## 1. Introduction

Three dimensional (3D) bioprinting is an emerging technology in regenerative medicine with ever increasing applications during the last decade [1,2]. The advantage of this technology is its ability to engineer in vitro tissues that better mimic the structural and functional complexity of native tissues [3,4]. Articular cartilage tissue engineering is one of the main applications of bioprinting, owing to the large interest in treating chondral defects, thereby preventing the development of osteoarthritis, or to create in vitro disease models for osteoarthritis. The structure of the articular cartilage tissue contains chemical, physical, and biological gradients that give rise to complex physical and mechanical properties [5]. Chondrocytes are surrounded by extracellular matrix (ECM) and their activities may be regulated by microenvironmental cues [6]. From a mechano-functional viewpoint, one of the most important characteristics of the articular cartilage matrix is its through-the-thickness variability of mechanical properties. Articular cartilage can be divided into three major zones, namely superficial, middle, and deep, each of which with different cell densities, compositions, and stiffness values [7]. Despite this heterogenous structure and well-characterized differences between the zones, homogenous scaffolds without any stiffness gradients are commonly used for cartilage regeneration [8]. Unsurprisingly, most such studies fail to regenerate functional, high-quality articular cartilage tissue. The same trend is observed in cartilage bioprinting where very few bioprinting strategies have been developed to recapitulate some of the gradients connecting the three zones in terms of cells or growth factors [9,10].

As far as stiffness gradients are concerned, most (bio)printing studies to date have focused on the large differences between cartilage and bone for osteochondral tissue engineering applications [11,12] but have often ignored the gradient within the cartilage itself. In non-printed scaffolds, strategies have focused on employing different biomaterial types, concentrations, or different crosslinking techniques. For instance, varying agarose concentrations between 3D printed layers from 2% to 3% led to zonal differences in the initial mechanical properties of the construct [13]. Stiffness gradients with Young’s moduli ranging between 1 kPa and 240 kPa were also obtained through controlled irradiation of an acrylamide/bis-acrylamide solution with various light doses and by moving a photo-mask at a controlled speed [14]. Additionally, recent research has investigated the use of visible light over UV light in photo-induced 3D bioprinting methods for tissue engineering, citing its enhanced biocompatibility [15]. Liu et al. have achieved successful stiffness gradients by varying the concentrations of polyethylene glycol (PEG)-precursor solutions to generate stiffness gradients in the range of 1–50 kPa [14]. Their results showed that softer hydrogels (Young’s modulus < 5 kPa) better support the deposition of cartilage-like matrix by bone marrow derived stem cells (MSCs) whereas stiffer hydrogels (Young’s modulus > 20 kPa) were more desirable for supporting chondrocyte-based cartilage deposition [16].

Although different strategies have been used to create stiffness gradients, there are still some challenges and limitations. It is widely recognized that the type, concentration, and crosslinking of bioinks influence their mechanical properties as well as the cell behavior [17,18]. Nevertheless, using different biomaterials or different concentrations of a single biomaterial to fabricate scaffolds with stiffness gradients makes it difficult to decouple the effect of stiffness from those pertaining to the presence of a specific biomaterial or its concentration. Moreover, when using different biomaterials with different stiffnesses, the constructs can face several challenges, such as the risk of delamination, the fabrication of less smooth stiffness gradients, and the need to optimize the 3D printing process for two different biomaterials.

The aim of this study was to fabricate a 3D bioprinted alginate-based scaffold with a stiffness gradient across its thickness by using a single biomaterial throughout the entire construct. To accomplish this, we developed a simple and novel approach based on directional ionic crosslinking. Following the successful generation of the stiffness gradient, the effects of such scaffolds on the cartilage matrix deposition by human articular cartilage-derived chondrocytes were assessed.

## 2. Materials and Methods

### 2.1. Cell Culture

Chondrocytes isolated from human articular cartilage of the knee were purchased from Lonza (NHAC-Kn, CC-2550, Lonza Bioscience, Oss, The Netherlands). The chondrocytes were obtained at passage 2 and were used at passage 4 in the following experiments. The cells were cultured in 75 cm^2^ tissue culture flasks at a density of 10.000 cells/cm^2^ and were fed with chondrocyte growth medium (CGM Bulletkit, CC-3216, Lonza Bioscience, The Netherlands) according to the manufacturer’s instructions. The CGM contains chondrocyte basal medium (CBM), R3-insulin-like growth factor-1 (R3-IGF-1), transferrin, insulin, human recombinant fibroblast growth factor-beta (hrFGF-β), fetal bovine serum (FBS), gentamicin and amphotericin-B (GA) (undisclosed concentrations by Lonza). The cells were cultured in an incubator at 37 °C, 90% humidity, and 5% CO_2_. The growth medium was refreshed twice weekly. Once the cells reached 80% confluency, they were detached with a trypsin/EDTA solution, collected by centrifugation, counted, and re-suspended in the medium for further expansion (passage 3) as before or for bioprinting (passage 4).

The cell-laden bioprinted scaffolds (preparation described in Section 2.2) were cultured in the chondrocyte differentiation medium (Bulletkit, CC-3225, Lonza Bioscience, The Netherlands) containing R3-IGF-1, transferrin, insulin, transforming growth factor-β1, FBS and GA (undisclosed concentrations by Lonza). 10 ng/mL transforming growth factor-β3 (SRP3171, Sigma-Aldrich, Delft, The Netherlands) and 70 mM L-ascorbic acid 2-phosphate (A8960, Sigma-Aldrich, Delft, The Netherlands) were added freshly to the differentiation medium for each medium change, according to the manufacturer’s instructions (TS-CC-112-7 02/20, Lonza). The chondrocyte differentiation medium was changed two times per week for 28 days using 2 mL of medium per scaffold. The scaffolds were kept in an incubator at 37 °C, 90% humidity, and 5% CO_2_. The experiment included three scaffolds per condition per time point (i.e., days 1, 7, 21, and 28). After expansion, chondrocytes were cultured in chondrogenic differentiation medium to counteract the dedifferentiation associated with monolayer expansion. According to the manufacturer’s instructions, this medium supports re-differentiation and enhances restoration of the chondrocyte phenotype, including matrix synthesis. Cells were maintained in this medium throughout the 3D culture period to ensure consistent phenotypic recovery.

### 2.2. 3D-Printing Process and Bioink Formulation

**Figure 1:**
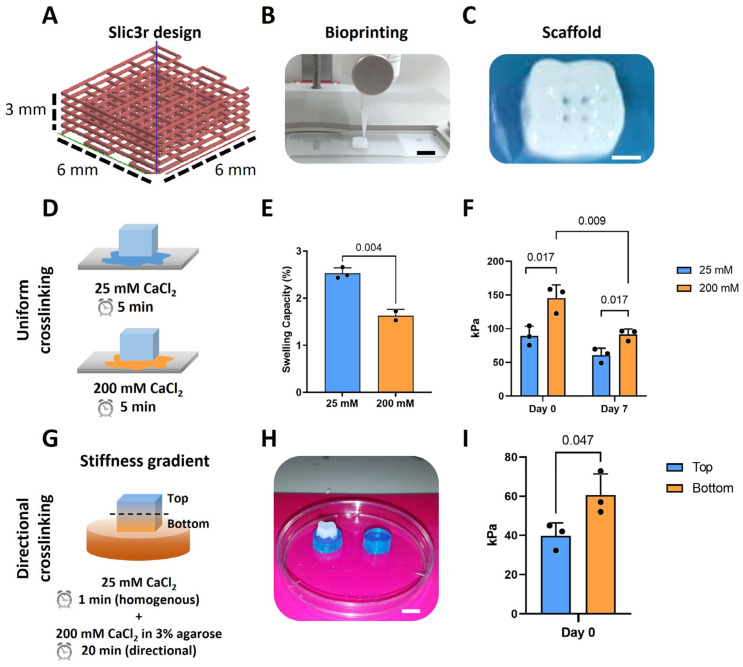
Directional ionic crosslinking can be used to generate scaffolds with a stiffness gradient. (**A**) Slic3r design of the scaffolds with dimensions of 6 × 6 × 3 mm^3^ and 12% infill density; (**B**) Printing of the scaffolds by using the BioX Cellink printer, scale bar: 6 mm; (**C**) Top view of the 3D printed scaffolds after ionic crosslinking showing the pores, scale bar: 2 mm; (**D**) A schematic representation of the ionic crosslinking of the scaffolds with 25 and 200 mM CaCl_2_; (**E**) Swelling capacity of the scaffolds crosslinked with 25 or 200 mM CaCl_2_ (*n* = 3); (**F**) Stiffness of the scaffolds crosslinked with 25 or 200 mM CaCl_2_ at day 0 and day 7 (*n* = 3); (**G**) A schematic representation of the directional crosslinking of the scaffolds to obtain a stiffness gradient; (**H**) Image of the scaffold placed on top of the blue agarose disk, scale bar: 6 mm; (**I**) Stiffness of the top and bottom zones of the scaffold after directional ionic crosslinking (*n* = 3).

A bioprinter (BIO-X, Cellink, Sweden) was used for the 3D (bio)printing of an alginate-based (bio)ink containing highly hydrated cellulose nanofibrils (CELLINK Bioink, Cellink, Sweden). When needed, the printer was UV-sterilized and used inside a cell culture hood to ensure the bioprinting process was running under sterile conditions. The (bio)ink was printed through a 27G (0.2 mm) conical nozzle with a speed of 4 mm/s. A pressure of ~14 kPa was selected for 3D (bio)printing, as described previously [6], to achieve high-precision scaffold fabrication and avoid possible cell damage. Briefly, a cube made in Solidworks 2020 with the dimensions of 6 × 6 × 3 mm^3^ (w × l × h) was sliced to obtain the desired pattern with a 12% infill (Figure 1A) using Slic3r (developed by A. Ranellucci). After (bio)printing, the scaffolds were ionically crosslinked with CaCl_2_ prior to further use (Section 2.3; Figure 1C).

The alginate-based ink (Cellink, Sweden) was mixed with the human chondrocytes as previously described [8]. Briefly, the alginate-based ink was taken from the commercial stock cartridge using a syringe. This ink was then mixed gently with the cell suspension (10:1) to make the bioink, using a sterile female–female luer–lock adaptor and a second syringe to ensure homogenization of the bioink. The bioink was then transferred to a new cartridge for carrying out the bioprinting. A final density of 5 × 10^6^ cells/mL was used for the bioprinting studies.

### 2.3. Crosslinking of 3D(Bio)Printed Constructs

Calcium chloride (CaCl_2_, C8106, Sigma-Aldrich, Delft, The Netherlands) was used as the ionic crosslinking agent. Three-dimensional prints were immersed in 1 mL of either 25 or 200 mM of CaCl_2_ for 5 min to fabricate the uniformly crosslinked scaffolds.

The scaffolds with a stiffness change throughout the construct were fabricated by using a novel methodology we developed to provide a directional ionic crosslinking. Briefly, the 3D printed constructs were crosslinked as follow: (1) post 3D (bio)printing, the constructs were immersed in 1 mL of 25 mM CaCl_2_ for 1 min to pre-crosslink the scaffold and ensure a minimum stability; (2) then the scaffolds were placed on top of disks made of routine use 3% agarose (A9539, Sigma-Aldrich, Delft, The Netherlands) containing 200 mM CaCl_2_, for 20 min (Figure 1G,H). The agarose disk had defined dimensions: 10 mm diameter and 5 mm height; to keep constant the number of ions to which the alginate hydrogel was exposed to. Due to the relatively long crosslinking time, the crosslinking process was performed in custom-made humid chambers within the biosafety cabinet at room temperature to prevent dehydration of the scaffolds, which could affect cell viability.

### 2.4. Swelling Capacity

To determine the swelling capacity, the constructs were soaked in fully supplemented chondrocyte differentiation medium for 24 h. The samples were dry blotted to remove the excess liquid and were weighed to obtain the equilibrium wet weight. Then, the constructs were freeze-dried and weighed to obtain the dry weight. The swelling capacity percentage was obtained by calculating the difference between the equilibrium wet weight and the dry weight the result of which was then divided by the dry weight:

$$\frac{W_{wet}-W_{dry}}{W_{dry}}\times 100$$

### 2.5. Mechanical Testing

Mechanical tests were performed using ESM303 motorized test stand (Mark 10, J.J.Bos BV, Gouda, The Netherlands) with a 2.5 N load cell. The test was performed up to a 30% unconfined compressive strain and a ramp displacement of 0.02 mm/s. The slope of the linear phase of the stress–strain curve from the compression tests was used to define the Young’s modulus.

### 2.6. Cell Viability Assay

To evaluate the viability of the bioprinted cells, live/dead staining (Live/Dead^®^ Viability/Cytotoxicity Kit, Thermo Fisher Scientific, The Netherlands) was performed at different time points. Each scaffold was cut into two equal parts from the middle. One part of the scaffolds was used for live/dead staining after 1, 7, 21, and 28 days of cell culture. Briefly, the cell-laden hydrogels were washed with 0.9% NaCl and were then incubated in 1 mL of a staining solution: 0.9% NaCl containing 5 mM calcein-AM (green, for live cells) and 2 mM ethidium homodimer-1 (red, for damaged/dead cells) for 30 min. After incubation, the constructs were rinsed again in 0.9% NaCl and were imaged under a fluorescence microscope (ZOE fluorescent cell imager, Bio-Rad, The Netherlands). Alive (green) and dead (red) cells were counted from four images per area, per scaffold (*n* = 3), per time point to assess cell viability.

### 2.7. Histological Staining

The other half of each scaffold (after 1, 7, 21 and 28 days) was fixed in 4% (*w*/*v*) paraformaldehyde (PFA) overnight at 4 °C with a tissue–fixative volume ratio of 1:20. The next day, the scaffolds were washed in 0.9% NaCl and were kept in a solution consisting of 50% ethanol and 45 mM CaCl_2_ for 1–2 days until they were further processed for histological analysis. The specimens were dehydrated for histological staining following serial dilutions of alcohol (70%, 80%, 90%, 100%) and xylenes before being embedded in paraffin. Thereafter, 6 μm thick slices were cut from the samples using a rotary microtome (HistoCore Biocut, Leica Microsystems, The Netherlands). The samples were deparaffinized and stained with hematoxylin and eosin (HHS32, HT110232, Sigma-Aldrich, The Netherlands) for cell morphology, with aldehyde fuchsin (P1528, Sigma-Aldrich, The Netherlands)/alcian blue (TMS-010-C, Sigma-Aldrich, The Netherlands) for sulfated glycosaminoglycan (sGAG) content, as previously described [19], and picrosirius red (365548, Sigma-Aldrich, The Netherlands) for collagen deposition. The stained samples were observed under a bright-field optical microscope (DM500 Leica Microsystems, The Netherlands). The histological images were equally divided into three zones along the gradient axis. Three biological specimens were measured to calculate the average value for each group. The number of cells was obtained through semiquantification of the different zones for each histological image. This number was normalized per unit area. In addition, the sGAG and collagen deposition was analysed on each slice where we compared day 1 vs. day 28 of culture. Briefly, ImageJ (v2.0.0, USA) was used to semi-quantify sGAG and collagen content by measuring the mean grayscale intensity of stained regions across different tissue layers (Top, Middle, Bottom) at various time points. The percentage of positive staining was determined using the formula:

(1) $$\frac{255-M}{255}\;\times \;100$$

where *M* is the Mean Gray Value and 255 is the maximum grayscale intensity in an 8-bit image. A lower Mean Gray Value indicates stronger staining, that can be correlated to a higher collagen or sGAG accumulation. The percentage of positive staining was calculated for each tissue section over time and plotted to assess changes in extracellular matrix deposition. For collagen and sGAG analysis, we used the images of day 1 to set a threshold as alginate stains light blue while sGAG stains darker blue/purple.

### 2.8. Statistical Analysis

The experiments were performed with a minimum of n of 3 specimens per group. All the results are expressed as mean ± standard deviation and results from individual specimens are represented in each graph with black dots. Statistical analysis (unpaired t test for individual data comparisons or two-way ANOVA for data containing multiple time points and layers) was performed using GraphPad Prism 8 (GraphPad Software, USA, Version 8.0.2). Probability *p*-values below 0.05 were considered statistically significant.

## 3. Results

### 3.1. Directional Ionic Crosslinking Enables Generation of Stiffness Gradients

Scaffolds of 6 × 6 × 3 mm^3^ and 12% infill density with specific pattern designs were 3D printed using the alginate-based ink (Figure 1A–C). The swelling of the scaffolds and the temporal variation in their stiffness when using different concentrations of CaCl_2_ were first investigated (Figure 1D–F). Increasing the CaCl_2_ concentration from 25 to 200 mM led to a significant decrease in the swelling capacity of the scaffolds from 2.65 to 1.6% (Figure 1E). The unconfined compression tests of these scaffolds revealed that the stiffness was significantly lower for the scaffolds crosslinked with the lower CaCl_2_ concentration of 25 mM (89.3 ± 14.5 kPa) as compared to the scaffolds crosslinked with the higher concentration of 200 mM (145.3 ± 19.7 kPa). After 7 days in culture medium, the printed scaffolds crosslinked with 25 mM CaCl_2_ experienced a loss in their stiffness of about 32.2%, while the scaffolds crosslinked with 200 mM experienced a loss of 37.2%. There was, though, still a significant difference between the stiffness of each group at day 7.

To obtain a stiffness gradient (within a single scaffold), the 3D printed scaffolds were firstly crosslinked with 25 mM CaCl_2_ for 1 min and were subsequently exposed to directional ionic crosslinking. The latter was achieved by placing the printed scaffolds on top of the agar disk (10Φ × 5h mm) containing 200 mM CaCl_2_ for 20 min (Figure 1G,H). This resulted in significantly different stiffnesses across the thickness of the constructs with values of 60.7 ± 10.9 kPa at the bottom of the scaffolds and of 39.8 ± 6.6 kPa at the top (Figure 1I).

### 3.2. The Stiffness Gradient of the Scaffolds Results in Zonal Cell Density and Matrix Deposition

**Figure 2:**
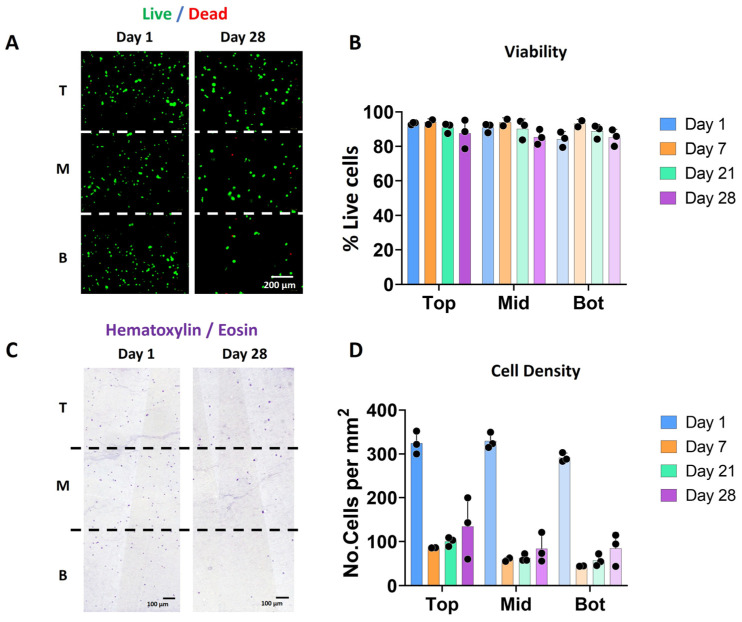
Scaffolds with a stiffness gradient show good chondrocyte viability over time. (**A**) Fluorescence images indicating cell viability based on live/dead staining (green: alive; red: dead) for days 1 and 28. The images correspond to the representative images of the three different sections in which the scaffold has been divided for analysis: T (top), M (middle) and B (Bottom). (**B**) Cell viability (in %) calculated from the live/dead stained images for days 1, 7, 21, and 28. (**C**) Bright-field microscopy images of the hematoxylin and eosin (H&E) staining showing cell distribution in the three different zones at days 1 and 28. (**D**) Quantification of the number of cells from the H&E staining at days 1, 7, 21, and 28 (*n* = 3).

The vast majority of the bioprinted cells were viable after 1 and 28 days of culture (Figure 2A,B) and no significant differences were observed in cell viability between the three different zones and on the different days (i.e., 93.4 ± 0.7, 91 ± 2.6, and 84.1 ± 4.4 on day 1 and 83.6 ± 8.4, 83.2 ± 4.3, and 82.9 ± 4.8 on day 28 for the top, middle and bottom zones, respectively). Nevertheless, less cells were visible in the scaffolds after 28 days of culture (Figure 2C,D).

Whereas Hematoxylin/eosin staining on day 1 showed a homogenous distribution of the cells throughout the entire scaffold, a decrease in cell density was observed over time in all the zones (Figure 2C). This reduction was more pronounced in the middle and bottom areas as compared to the top of the scaffolds. This decrease in cell number was observed starting on day 7 and was not apparently related to an evident decrease in viability (see Figure 2B). Further experiments may clarify if this decrease can be associated with cell migration or even related to the bioprinting process.

**Figure 3:**
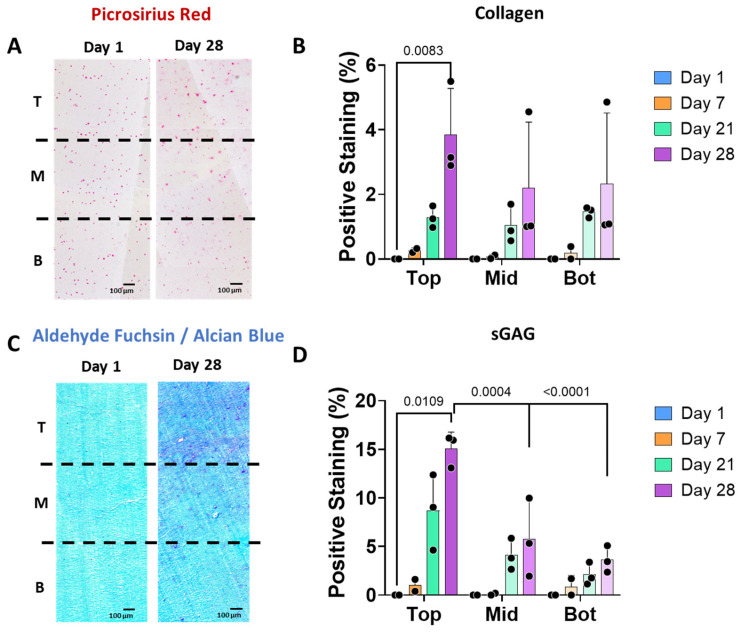
The stiffness gradient regulates cartilage-like matrix deposition by human chondrocytes. (**A**) Histological images of picrosirius red staining showing collagen presence in the three different zones of the scaffolds: T (top), M (middle) and B (Bottom) at days 1 and 28. (**B**) Collagen stained areas at days 1, 7, 21, and 28, where background staining from day 1 has been subtracted in all time points as normalization step. (**C**) Alcian blue staining showing background staining of day 0 in light blue and the sGAG deposition in dark blue of the three different zones at day 28. (**D**) sGAG stained areas at days 1, 7, 21, and 28 (*n* = 3), where background staining from day 1 has been subtracted in all time points as normalization step.

The scaffolds were analysed for collagen and sGAG deposition in the different zones and overtime (Figure 3). Overall, there was a limited amount of collagen or sGAG produced over time, perhaps due to the nature of our hydrogel (mainly alginate). Positive staining for picrosirius red staining was localized surrounding the chondrocytes cells (Figure 3A,B), where a significant increase in staining was observed in the top zone after 28 days. Alcian blue staining showed background staining of our alginate hydrogel in light blue at day 0, and sGAG deposited in dark blue/purple throughout the construct. Positive staining for sGAG was observed after 28 days in all zones. There was a significant increase in sGAG in the top zone as compared to the middle and bottom zones of the scaffolds after 28 days of culture (Figure 3C,D).

## 4. Discussion

The development of scaffolds that mimic the biological and mechanical gradients of native tissues is of great importance for the study of (patho)physiology and could aid in the rational design of scaffolds for tissue regeneration. These gradients may direct the cells to generate cartilage that mimics the natural gradient. In this study, a novel directional ionic crosslinking method was established to engineer 3D bioprinted scaffolds with a stiffness gradient by using a single biomaterial with a single concentration. After successful fabrication of the gradient hydrogels, chondrocytes were incorporated into the bioink and were bioprinted. Then, the effects of the stiffness gradient on cell viability, cell number, and matrix deposition (sGAG and collagen) were studied in the constructs during 4 weeks of culture. Human chondrocytes embedded in the bioprinted construct were observed to maintain their viability over a period of 28 days. Interestingly, a gradient in the cell number and sGAG deposition was observed after 28 days with more cells and sGAG being present in the softer zones of the scaffolds (at the top) as compared to the stiffer ones (at the bottom).

The experiments performed with homogeneous scaffolds demonstrated that increasing the crosslinking agent concentration resulted in an increase in the stiffness and a decrease in the swelling of the printed constructs. However, in the scaffolds with a stiffness gradient, no difference in the swelling was observed between the bottom and top zones as the shape of the hydrogels was not altered. This may be associated with the lower crosslinking rate of the gradient scaffolds as determined by the directional diffusion of the CaCl_2_ solution from the agar disk through the scaffolds. The slower crosslinking has been previously linked to a lower shrinkage of the alginate [20,21], which could reduce the difference in the swelling properties throughout the gradient scaffold. Because ion diffusion is highly sensitive to sample geometry, filament thickness, and overall volume, crosslinking conditions cannot be directly transferred between constructs of different sizes. Future work could incorporate diffusion modelling to establish a more generalizable framework for predicting crosslinking kinetics across varying scaffold designs.

After one week of immersion in culture medium, the stiffness of the homogeneous scaffolds decreased by ~30%. This is thought to have been caused by the leaching of calcium ions from the scaffolds to the surrounding environment, as previously observed in other alginate systems [22], as well as by the presence of potential calcium chelators such as phosphate [23], or competing ions such as Zn^2+^ and Mg^2+^ [24], which are present in the culture medium and may influence calcium dynamics [20,25]. The latter compete with calcium for the negatively charged alginate but cannot form similarly strong crosslinking bonds with alginate. The percentage decrease in stiffness is relatively similar between groups (32–37%). The slightly higher loss in the more crosslinked scaffolds may reflect the minimum amount of calcium that alginate can retain once exposed to culture medium. The lower-crosslinked scaffold is likely to reach this minimal crosslinking threshold sooner, meaning that it has less remaining stiffness to lose, whereas the more crosslinked scaffold can continue to undergo partial decrosslinking before reaching the same plateau. These results highlight that the currently optimized medium to maintain cell growth is not optimized yet for maintaining the mechanical stability of the bioprinted alginate-derived hydrogels. From our results, it can be inferred that the crosslinking gradient does not remain fully stable over time, although the proportional differences between conditions are preserved.

Using gradient hydrogels, we can determine how stiffness gradients affect cell proliferation and cartilage formation by chondrocytes in a single construct. The stiffness gradient generated in the alginate-based scaffolds (i.e., 20 kPa difference, from about 60 kPa at the bottom to 40 kPa at the top of the scaffolds) did not affect the viability of human chondrocytes over the 28 days of culture, and a higher cell density was observed in the softer zone from day 7, which could be explained by a higher proliferation rate in this zone. In general, most studies working with constructs or scaffolds to engineer articular cartilage have demonstrated a clear difference between cell behavior in soft and stiff biomaterials where softer hydrogels are generally found to lead to a higher cell proliferation than stiffer hydrogels (with the same composition as the soft hydrogels). Previous studies working with different biomaterials, such as RGD-modified agarose [6] or PEG-based [26] photo-crosslinked gradient scaffolds with similar stiffness values in the ranges of 2–53 and 5–60 kPa, respectively, have also observed an increase in the proliferation of chondrocytes in the soft zone as compared to the stiff zone. Previous studies have shown a reduction in the cell number and chondrogenic phenotype when using a PEG-based hydrogel with a modulus gradient of more than 13.1 kPa; however, a lower storage modulus preserved the cell number and phenotype [27]. It has been suggested that cell proliferation could be initially inhibited by the higher physical constraint of the stiffer microenvironment [26]. Stiffness gradients have also been combined with growth factor gradients to stimulate the differentiation of stem cells towards bone or cartilage-like phenotypes. The stiffness gradient was achieved by photocrosslinking glycol chitosan hydrogels microparticles with two different degrees of methacrylation, while the biochemical gradient was achieved by mixing both TGF-β1 and BMP-2 growth factors in the hydrogel, before crosslinking [15,28]. Other cell types such as MSCs have also been used, in the context of chondrogenesis, in combination with alginate-based hydrogels containing a gradient of stiffness [29]. In this study, the authors also observed a significant increase in cell proliferation (measured by DNA content) with softer hydrogels (10 kPa) compared to higher stiffness (from 50 kPa).

In addition to the increased cell density, the softer zone was observed to have a higher cell-derived matrix deposition as compared to the stiffer zone. This was not observed in a uniformly crosslinked alginate hydrogel (90 mM CaCl_2_ for 2 min, which falls in between the two concentrations of CaCl_2_ studied here) with similar bulk mechanical properties to the stiffness gradient hydrogel, as we have shown in our previous work [9]. It is noteworthy to mention that articular cartilage does not have a lot of sGAG in the top zone, thus our system probably needs to integrate an additional cell density gradient that can deliver higher content of sGAG in the middle zone, as we have previously shown [9]. To the best of our knowledge, no studies have investigated this in alginate-derived hydrogels with stiffness gradients, but our results are in agreement with previous studies that have also observed a higher matrix deposition in softer alginate-derived hydrogels as compared to stiffer gels. In addition, previous research [6] has demonstrated that a RGD-functionalized agarose-based hydrogel with a stiffness gradient in the range of 2–53 kPa promotes the deposition of significantly higher amounts of collagen and GAGs in the soft zones of the hydrogel. Another study in which chondrocytes were cultured in fibrin hydrogels with a Young’s modulus of 1–30 kPa found enhanced synthesis of ECM constituents, such as sGAG and collagen type II, in fibrin hydrogels with a lower stiffness [30]. These studies together with our results show the significant impact of mechanical properties on modulating the behavior of human chondrocytes and regulating tissue formation. In our study, the stiffness range of the gradient scaffolds is below the mechanical properties of the native articular cartilage (400–800 kPa) [31]. Even though soft gels do not mimic the mechanical properties of native tissues, this research and others demonstrated that even small differences in stiffness can have a significant impact in cell number and matrix content, suggesting that the development of soft substrates simulating the mechanical properties of developing cartilage may represent a promising strategy for cartilage tissue regeneration [32]. The effect of stiffness on chondrocyte proliferation is intertwined with factors such as crosslinking level, pore size, and matrix density, all of which influence nutrient transport within the hydrogel. These aspects should be taken into consideration when interpreting studies that aim to isolate the role of stiffness on cell growth [33].

## 5. Conclusions

In this study, scaffolds with a stiffness gradient were fabricated using a single type and concentration of biomaterial treated with the novel and simple technique of directional ionic crosslinking. This technique can overcome the disadvantages associated with the use of different types and/or amounts of biomaterials to modulate the mechanical properties of a regenerative construct. Our results showed that the softer zones of the hydrogel promote a higher cell density and cartilage-like matrix deposition by chondrocytes as compared to the stiffer zones after 28 days of culture. To further mimic the properties of developing articular cartilage, further research is needed to investigate the effects of combining stiffness gradients with cell gradients for cartilage tissue engineering applications.

## Data Availability

The original contributions presented in the study are included in the article, further inquiries can be directed to the corresponding author.

## Abbreviations

The following abbreviations are used in this manuscript:

3D	Three-dimensional
ECM	Extracellular matrix
MSC	Mesenchymal stem cell
CGM	Chondrocyte Growth Medium
CBM	Chondrocyte Basal Medium
